# Validation of red cell distribution width as a COVID-19 severity screening tool

**DOI:** 10.2144/fsoa-2020-0199

**Published:** 2021-04-20

**Authors:** Mandana Pouladzadeh, Mehdi Safdarian, Parastoo Moradi Choghakabodi, Fatemeh Amini, Alireza Sokooti

**Affiliations:** 1 Department of Emergency Medicine, School of Medicine, Ahvaz Jundishapur University of Medical Sciences, Ahvaz, 61357-15794, Iran; 2 Nanotechnology Research Center, Ahvaz Jundishapur University of Medical Sciences, Ahvaz, 61357-15794, Iran; 3 Thalassemia & Hemoglobinopathy Research Center, Health Research Institute, Ahvaz Jundishapur University of Medical Sciences, Ahvaz, 61357-15794, Iran; 4 Department of Persian medicine, School of Medicine, Shiraz University of Medical Sciences, Ahvaz, 61357-15794, Iran; 5 Department of Pathology, Razi Hospital, School of Medicine, Ahvaz Jundishapur University of Medical Sciences, Ahvaz, 61357-15794, Iran

**Keywords:** COVID-19, mortality, RDW-SD, red cell distribution width

## Abstract

**Aim:**

The aim of this study is the predictive validation of red cell distribution width (RDW) in COVID-19 patients.

**Method:**

In total, 331 COVID-19 patients were classified as ‘severe’ and ‘nonsevere’ groups based on the WHO standard criteria. The levels of RDW standard deviation (SD) were evaluated as both continuous and categorical variables. Multivariate statistical analyses were used.

**Results:**

RDW-SD ≤43 and ≤47 fl thresholds showed high specificity (90.1–91.4%) for diagnosing nonsevere illness and no risk of death. RDW-SD >47 indicated severe illness and a high mortality risk while 43<RDW-SD≤47 indicated severe illness with low risk of death.

**Conclusion:**

RDW-SD levels may be a potent independent predictor of the infection severity and mortality probability in COVID-19 patients.

## Lay abstract

The red cell distribution width (RDW) test is part of a complete blood count, and calculates the difference in size from the largest to the smallest red blood cell. Our analysis of 331 COVID-19 patients showed that the RDW standard deviation index (RDW-SD) has the potential to differentiate nonsevere from severe patients as well as predict the death of COVID-19 patients. In this regard, RDW-SD ≤47 fl indicated nonsevere illness and low risk of death while RDW-SD >47 indicated severe illness and a high risk of death. Also, 43< RDW-SD ≤47 indicated severe illness with low risk of death.

## Graphical abstract

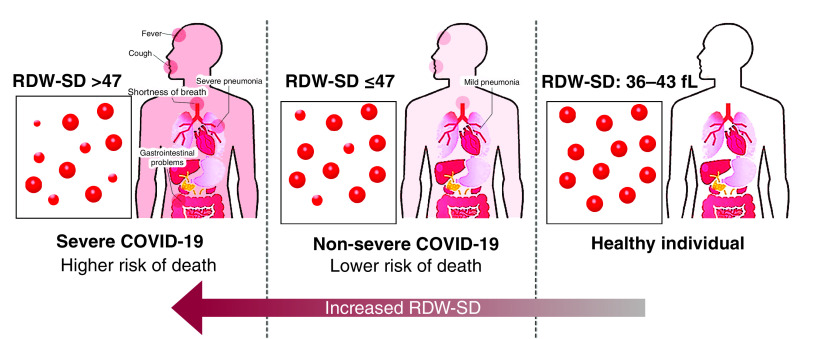

COVID-19 infection, caused by SARS-CoV-2, has become a serious threat to healthcare systems globally [1]. A large number of clinical studies are currently underway to find diagnostic, prognostic, therapeutic and prophylactic agents. However, time is the main restrictive factor in public health emergency preparedness (PHEP). Therefore, timely discovery of diagnostic and/or prognostic factors related to COVID-19 pathophysiology can be useful in tracking the severity and outcomes of the disease, appropriate treatment as well as the discovery of effective therapeutic agents.

The significant role of erythrocytes in the pathophysiology of COVID-19 was pointed out in a few recent reports. The standard deviation of red blood cell distribution width (RDW-SD) and the coefficient of variation of red blood cell distribution width (RDW-CV) may be a predictor of the severity of COVID-19 [2,3]. RDW is a numerical measure of the variability of the circulating erythrocyte volume. An elevated RDW implicates an increased rate of red blood cell (RBC) destruction, dysfunctional erythropoiesis and or shortened RBC lifespan [4]. Various researches have confirmed the predictive role of RDW for the clinical outcome of respiratory and/or cardiovascular disorders, such as chronic obstructive pulmonary disease (COPD) [5,6], pulmonary embolism [7], community-acquired pneumonia (CAP) [8], heart failure (HF) [9] and acute myocardial infarction (AMI) [10]. In addition, RDW is a good predictive index of the clinical outcomes and mortality in pulmonary arterial hypertension patients [6,11,12].

Elevated RDW is correlated with suppressed erythrocyte turnover indicating erythropoietin distress. Suppressed erythrocyte turnover may play as a compensatory mechanism to maintain circulating RBCs [13]. RBC count was decreased in rhesus macaques infected with SARS-CoV-2 [14]. In this regard, the size of the spleen is significantly reduced in autopsied dead COVID-19 patients due to the discharge of erythrocytes from the spleen into circulation as a normal physiological response to anemia [15]. SARS-CoV-2 spike protein binds to the CD147 receptor that is one of the main receptors for mediating SARS-CoV-2 invasion and virus diffusion among other cells; the CD147 receptor is also determinative of the OK blood group system [16,17]. The optimized recirculation of erythrocytes is repealed following the blockage of CD147; this leads to the elective trapping of RBCs in the spleen and eventually, the progression of anemia [18].

Based on Topaz *et al.*‘s study, RDW >14.5% is a predictive index of poor outcomes in hospitalized influenza patients [19]. Also, in a recent report from China, elevated RDW was associated with severe COVID-19 [3]. In this prospective analytical study, the prognostic value of both RDW-SD and RDW-CV was evaluated in COVID-19 cases.

## Method

### Study design

This prospective analytical study was conducted on 402 COVID-19 patients referred to the Emergency Department of Razi Hospital in Ahvaz from 3 April 2020 to 20 May 2020. The clinical and paraclinical characteristics were evaluated, and laboratory tests were performed in the in-hospital laboratory using standard kits (Merck KGaA, Darmstadt, Germany). Data were collected from medical records on a daily basis.

For analysis, COVID-19 patients were classified into ‘severe’ and ‘nonsevere’ groups based on the following WHO criteria [20]:

- Severe acute distress syndrome: PaO2/FiO_2_ ≤100 mmHg;
- Dramatically elevated counts of WBCs and neutrophils, and lymphocyte depletion;
- Remarkable cytokine storm;
- Multiple organ failure;
- Prolonged hospitalization (>7 days);
- Dyspnea and fatigue and sometimes diarrhea and anorexia;
- Severe lung lobes involvement in CT scan: scores 3 to 4 (51–100%) and their clinical and paraclinical characteristics assessed.

All patients were followed up during the hospitalization as well as by telephone 2 months after discharge. For assessment of the prognostic value of RDW levels, prognostic receiver operating characteristic curves (ROC) of RDW, Harrell's C-Index and prognostic accuracy were detected.

Healthy controls comprised family members of each patient who referred to the emergency room; they tended to do a CBC test for routine check-up.

### Statistical analysis

SPSS 22 statistical software (SPSS, Inc., IL, USA) was used for statistical analysis. Based on the results of the Kolmogorov–Smirnov test, variables were normally distributed. Categorical variables were compared using the chi-square test and presented as frequency and percentages, while continuous variables were compared using the independent-samples *t*-test and presented as the mean ± SD (standard deviation). For assessment of the prognostic value of RDW levels, receiver operating characteristic curves (ROC) was used to determine the cutting point and its sensitivity and specificity. The accuracy formula was used to determine the efficiency of the index.

Associations and correlations of RDW levels with continuous variables were assessed by Pearson's correlation coefficient. The multivariable Cox proportional hazards model was appropriate to identify independent prognostic factors. P-value < 0.05 was regarded as statistically significant.

## Results

Among 402 COVID-19 patients hospitalized during the study period (included more than 28 days follow-up), 71 cases were excluded from the analysis due to incomplete data. A total of 331 COVID-19 patients were analyzed. Respectively, 178 (53.77%) men and 153 (46.23%) women with mean age 55.27 ± 16.48 and 54.88 ± 16.27 years were analyzed (Table 1). The mean age of patients with severe COVID-19 (63.10 ± 16.20 years) was greater than nonsevere COVID-19 patients (51.98 ± 15.49 years). COVID-19 patients were classified as severe and nonsevere groups as detailed above and their characteristics described in Table 1.

**Table 1.** Demographic, clinical and laboratory characteristics of COVID-19 patients.

Characteristics	Nonsevere cases (n = 233)	Severe cases (n = 98)	p-value	All cases (n = 331)
Age (years) Total Male Female	Mean ± SD 51.98 ± 15.49 51.29 ± 15.10 52.73 ± 15.94	Mean ± SD 63.10 ± 16.20 64.50 ± 16.51 61.14 ± 15.75	^†^	Mean ± SD 55.38 ± 16.65 54.88 ± 16.27
	n (%)	n (%)	p-value	n (%)
Gender Male Female	121 (51.93) 112 (48.07)	57 (58.16) 41 (41.84)	0.33	178 (53.77) 153 (46.23)
Basic disease: Total HTN DM	120 (51.50) 32 (13.70) 58 (24.90)	82 (83.67) 15 (15.30) 37 (37.80)	^†^ 0.70 0.01	202 (61.03) 47 (14.20) 95 (28.70)
Mortality total (n = 40) No disease (n = 138) HTN (n = 47) DM (n = 95) RDW-SD >47 (n = 44) RDW-CV >15% (58)	0/233 (0) 0/119 (0) 0/32 (0) 0/58 (0) 0/10 (0) 0/28 (0)	40/98 (40.816) 11/19 (57.90) 3/15 (20) 13/37 (35.10) 19/34 (55.90) 13/30 (43.30)	^†^ ^†^ 0.02 ^†^ ^†^ ^†^	40 (12.10) 11/138 (8) 3/47 (0.06) 13/95 (13.60) 19/44 (43.20) 13/58 (22.40)
RDW-SD ≤47 (normal) >47	223 (95.70) 10 (4.30)	64 (65.30) 34 (34.70)	^†^	287 (86.70) 44 (13.30)
RDW-CV ≤15 (normal) >15	205 (88) 28 (12)	68 (69.40) 30 (30.60)	^†^	273 (82.50) 58 (17.50)
Symptoms				
Fever	177 (75.97)	92 (93.88)	^†^	269 (81.27)
Dry cough	126 (54.08)	87 (88.78)	^†^	213 (64.35)
Fatigue	108 (46.35)	94 (95.92)	^†^	202 (61.03)
Sputum production	103 (44.20)	87 (88.78)	^†^	190 (57.40)
Loss of smell	33 (14.16)	62 (63.27)	^†^	95 (28.70)
Shortness of breath	41 (17.60)	57 (58.16)	^†^	98 (29.60)
Sore throat	16 (6.87)	39 (39.80)	^†^	55 (16.62)
Muscle or joint pain	46 (19.7)	78 (79.6)	^†^	124 (37.50)
Headache	18 (7.7)	56 (57.1)	^†^	74 (22.4)
Diarrhea	15 (6.44)	5 (5.1)	0.64	20 (6)
Laboratory test	Nonsevere cases Mean ± SD	Severe cases Mean ± SD	p-value	Mean ± SD
WBC (10*3 μl)	7.11 ± 3.50	11.09 ± 7.81	^†^	8.29 ± 5.47
RBC (μl)	4.35 ± 0.62	4.04 ± 0.74	^†^	4.26 ± 0.67
Neutrophils (%) of the total WBC	66.40 ± 11.011	76.81 ± 11.15	^†^	69.49 ± 12.02
Lymphocytes (%) of the total WBC	27.08 ± 10.63	16.10 ± 9.67	^†^	23.83 ± 11.50
Hb (g/dl)	12.50 ± 1.90	11.56 ± 1.94	^†^	12.22 ± 1.96
Hct (%)	36.02 ± 5.28	33.70 ± 5.67	^†^	35.33 ± 5.49
MCV (Fl)	82.91 ± 6.98	84.05 ± 8.22	0.19	83.25 ± 7.37
MCHC (g/dl)	34.66 ± 1.42	34.22 ± 1.43	0.01	34.53 ± 1.43
RDW-CV (%)	13.80 ± 2.60	14.71 ± 2.48	^‡^	14.07 ± 2.60
RDW-SD (fl)	37.95 ± 5.95	46 ± 7.56	^†^	40.34 ± 7.43
ESR (mm/hr)	40.96 ± 25.11	50.33 ± 27	^‡^	43.74 ± 26

† p-value < 0.001.

‡ p-value < 0.01.

DM: Diabetes mellitus; ESR: Erythrocyte sedimentation rate; HTN: Hypertension; MCHC: Mean corpuscular hemoglobin concentration; MCV: Mean corpuscular volume; RBC: Red blood cell; RDW-CV: Coefficient of variation of red blood cell distribution width; RDW-SD: Standard deviation of red blood cell distribution width; WBC: White blood cell.

The range of RDW-CV and RDW-SD values for healthy people without any underlying diseases and coronavirus disease referred to the emergency department of Ahvaz hospitals during the recent epidemic were 11.5–15% and 36–43 fl, respectively. Based on a statistical comparison of clinical and laboratory markers between nonsevere and severe COVID-19 patients, the following results were obtained (Table 1):

- The frequency (%) of COVID-19 symptoms including fever, dry cough, fatigue, sputum production, loss of smell, shortness of breath, muscle or joint pain, sore throat and headache was significantly higher in severe patients than nonsevere patients (p = 0.0001). The most common symptom in patients with COVID 19 was fever (81.27%), which was 75.97% in the nonsevere group and 93.88% in the severe group. However, fatigue was also common in the severe group with 95.92%;
- The mean levels of WBCs, RDW-CV, RDW-SD, PDW, MCHC and ESR were significantly higher in severe patients compared with nonsevere patients (p = 0.0035);
- The percentage of neutrophils was significantly higher in severe patients compared with nonsevere patients (p = 0.0001) as it has progressed to neutrophilia;
- The percentage of lymphocytes was significantly lower in severe patients compared with nonsevere patients (p = 0.0001), and lymphopenia in severe patients was significantly more evident;
- The mean levels of RBC, Hb and Hct were significantly lower in severe patients compared with nonsevere patients (p = 0.0001);
- The mean level of bicarbonate (HCO_3_-act) was significantly lower in severe patients (25.17 ± 6.87) than nonsevere patients (27 ± 5.52), indicating a progression of metabolic acidosis in severe patients. Other respiratory indicators include PH, pCO_2_, pO_2_, Temp, FiO_2_ and HCO_3_-std were not significantly different;
- The levels of renal biomarkers, including AST, direct bilirubin and BUN (serum) were significantly higher in severe COVID-19 patients than nonsevere COVID-19 patients (p < 0.01);
- The mean length of in-hospital stay (day) was higher in severe patients compared with nonsevere patients, but not significantly so (p = 0.10);
- The prevalence (%) of basic diseases was significantly higher in severe patients compared with nonsevere patients (p = 0.0001).

**Figure 1.**
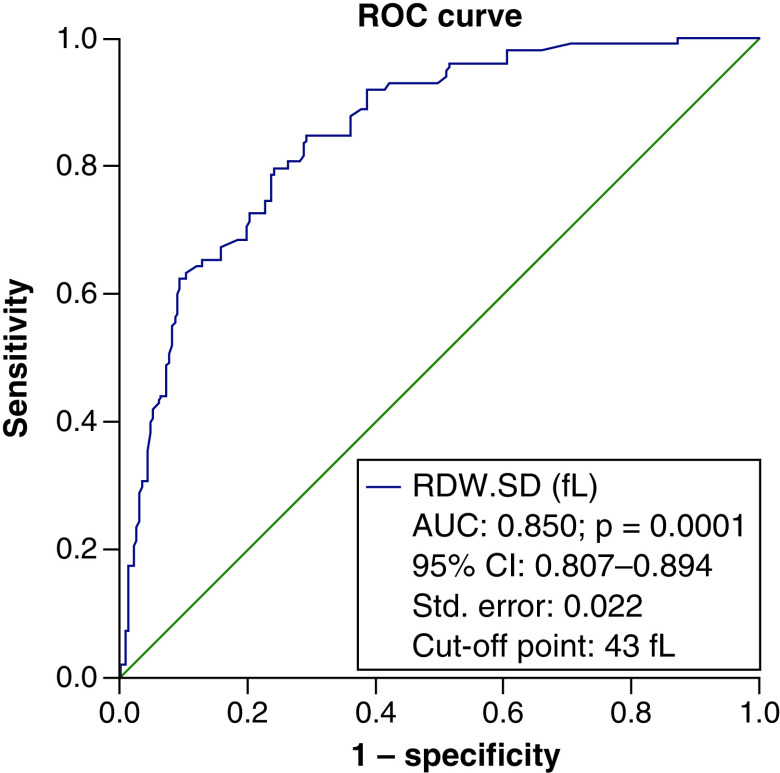
The ROC curve to determine the efficacy of red cell distribution width-standard deviation in identifying the disease severity in COVID-19 patients. The specificity and the sensitivity of the cut-off point of 43 were respectively 90.1 and 62.2% with an accuracy of 81%. Diagonal segments are produced by ties.

**Figure 2.**
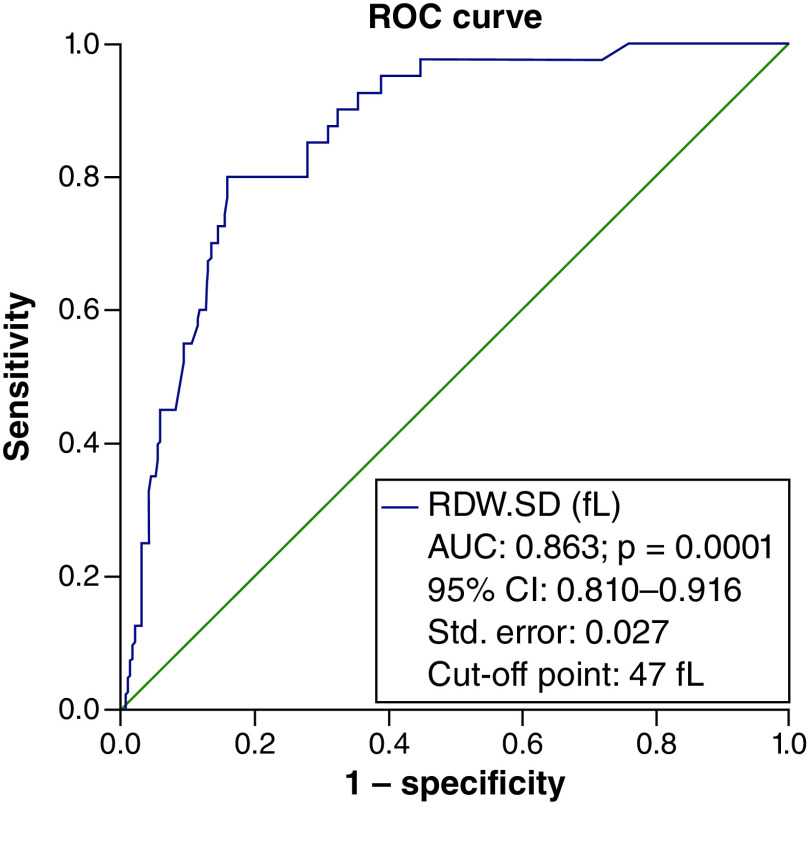
The ROC curve to determine the efficacy of red cell distribution width-standard deviation in calculating the probability of death in COVID-19 patients. The specificity and the sensitivity of the cut-off point of 47 were respectively 91.4 and 47.5% with an accuracy of 86%. Diagonal segments are produced by ties.

To determine the efficacy of RDW-SD in identifying the severity of the disease in people with COVID-19, an ROC curve was used, which resulted in an area below the curve of 85% and a cut-off point of 43 considered. The specificity of this point was 90.1% and the sensitivity was 62.2% with an accuracy of 81% (Figure 1). Again, the ROC curve was considered to determine the RDW-SD efficiency in calculating the probability of death of patients. The cut-off point was considered to be 47. The area under the curve was (AUC = 0.863) with a specificity of 91.4%, a sensitivity of 47.5% and an accuracy of 86% (Figure 2).

### Associations of RDW levels with clinical & laboratory variables

Results of linear and/or logistic regression (simple and multiple) and Pearson's correlation coefficient analysis showed that there was a significant association between RDW-SD (fl) levels and a series of biomarkers, symptoms, the severity of disease and mortality (p < 0.05) as illustrated in Table 2 & Supplementary Tables 1 & 2. However, there was no significant correlation between RDW-SD levels and LOS (day), and some biomarkers (p > 0.05).

**Table 2.** Associations and correlations of red cell distribution width-standard deviation (fl) and coefficient of variation of red blood cell distribution width (%) levels with demographic, clinical and laboratory variables.

Variable	RDW-SD (fl)			RDW-CV (%)		
	r	95% CI	p-value	r	95% CI	p-value
Age	0.3348	0.235–0.427	^†^	0.1597	0.0528–0.2630	^‡^
WBC (10*3 μl)	0.2398	0.135–0.339	^†^	0.1384	0.03105–0.2426	0.01
RBC (μl)	-0.4652	-0.545–-0.376	^†^	-0.2146	-0.3151–-0.1093	^†^
Neutrophils (%)	0.1478	0.0404–0.251	^‡^	0.0506	-0.0576–0.1577	0.35
Lymphocytes (%)	-0.2015	-0.302–-0.0957	^†^	-0.0510	-0.1580–0.0570	0.35
Hb (g/dl)	-0.3584	-0.449–-0.260	^†^	-0.3736	-0.4628–-0.2770	^†^
Hct (%)	-0.3048	-0.399–-0.203	^†^	-0.3183	-0.4120–-0.2180	^†^
MCV (Fl)	0.3436	0.2448–0.4353	^†^	-0.1556	-0.2591–-0.0486	^‡^
MCH (pg)	0.2036	0.09795–0.3048	^†^	-0.2344	-0.3337–-0.1298	^†^
MCHC (g/dl)	-0.2486	-0.3471–-0.1447	^†^	-0.2737	-0.3706–-0.1709	^†^
RDW-CV (%)	0.5301	0.4479–0.6035	^†^	–	–	_
MPV (fl)	0.0378	-0.0702–0.1451	0.49	0.1610	0.05414–0.2642	^‡^
PDW (%)	0.0084	-0.099–0.116	0.87	0.1900	0.0839–0.2919	^†^
PLT (10*3/ul)	-0.1112	-0.216–-0.00336	0.04^ps^	-0.0521	-0.1590–0.05599	0.34
ESR (mm/h)	0.1453	0.0380–0.249	^‡^	-0.0202	-0.1278–0.0877	0.71
AST (unit/l)	0.1281	0.0205–0.232	0.01	0.0416	-0.0664–0.1488	0.45
ALT (unit/l)	0.02133	-0.0867–0.129	0.69	-0.0024	-0.1102–0.1054	0.96
Direct bilirubin (mg/dl)	0.2817	0.1794–0.3781	^†^	0.2241	0.1192–0.3241	^†^
Total bilirubin (mg/dl)	0.1100	0.0022–0.2153	0.04^ps^	0.07012	-0.0379–0.17661	0.20
BUN serum (mg/dl)	0.3227	0.2226–0.4161	^†^	0.1880	0.0818–0.2899	^†^
Creatinine (mg/dl)	0.1859	0.0797–0.2880	^†^	0.1094	0.0016–0.2147	0.04
LDH (unit/l)	0.1587	0.05180–0.2621	^‡^	0.04619	-0.0619–0.1532	0.40
Plasma (BS) mg/dl	-0.1097	-0.2150–-0.00193	0.04^ps^	-0.0540	-0.1608–0.0541	0.32
VBG (PH)	-0.0893	-0.1952–0.01872	0.1	-0.0703	-0.1768–0.0377	0.20
VBG (PCO_2_)	-0.01432	-0.1220–0.0936	0.79	0.0433	-0.0647–0.1505	0.43
VBG (PO_2_)	0.0998	-0.008–0.2054	0.07	0.0941	-0.0138–0.1999	0.08
VBG (HCO_3_-act)	-0.0684	-0.1750–0.0396	0.21	-0.01704	-0.1246–0.0909	0.75
VBG (HCO_3_-std)	-0.0169	-0.1246–0.0910	0.75	-0.01267	-0.1203–0.0952	0.81
BE	-0.0726	-0.1791–0.0354	0.18	-0.0475	-0.1545–0.0605	0.38
BB	-0.0683	-0.1749–0.0397	0.21	-0.0487	-0.1557–0.0594	0.37
Length of in-hospital stay (day)	0.0822	-0.0258–0.1884	0.13	0.0290	-0.0790–0.1364	0.60

† p-value < 0.001.

‡ p-value < 0.01.

ALT: Alanine aminotransferase; AST: Aspartate aminotransferase; BB: Buffer Base; BS: Blood sugar; BUN: Blood urea nitrogen; DM: Diabetes mellitus; ESR: Erythrocyte sedimentation rate; Hb: Hemoglobin; Hct: Hematocrit; HTN: Hypertension; LDH: Lactate dehydrogenase; MCHC: Mean corpuscular hemoglobin concentration; MCV: Mean corpuscular volume; MPV: mean platelet volume; PLT: Platelet count; ps: Partially significant; RBC: Red blood cell; RDW-CV: Coefficient of variation of red blood cell distribution width; RDW-SD: Standard deviation of red blood cell distribution width; VBG: Venous blood gases; WBC: White blood cell.

Based on the univariate logistic regression analysis, age and RDW-SD (fl) levels had a significant association with several clinical signs and outcomes of disease (p < 0.05) while RDW-CV % showed no significant relation with the clinical signs (p > 0.05). Subsequently, a multivariate logistic regression analysis of 331 COVID-19 patients was performed for clinical signs and outcomes (categorical variables) based on RDW-SD (fl) and age (Supplementary Tables 1 & 2). The results showed that RDW-SD (fl) levels can significantly predict primary and secondary outcomes in COVID-19 patients, but RDW-CV % did not show a strong association with the disease severity and survival status (Supplementary Table 2). Overall, RDW-CV % may not be a potential predictor of outcomes.

Pearson correlation coefficient was used to evaluate the relationship between RDW-SD and RDW-CV with the length of in-hospital stay. The values were r = 0.08 (p = 0.12) and r = 0.03 (p = 0.58), respectively; these correlations were not significant (Table 2). The AUC was 0.64 for determining the sensitivity and specificity of RDW-CV in predicting the severity of COVID-19. Also for predicting death, this level was low (AUC = 0.699; 64%), which shows a weak relation with both variables.

### Association of RDW-SD with survival probability & severity of disease

**Figure 3.**
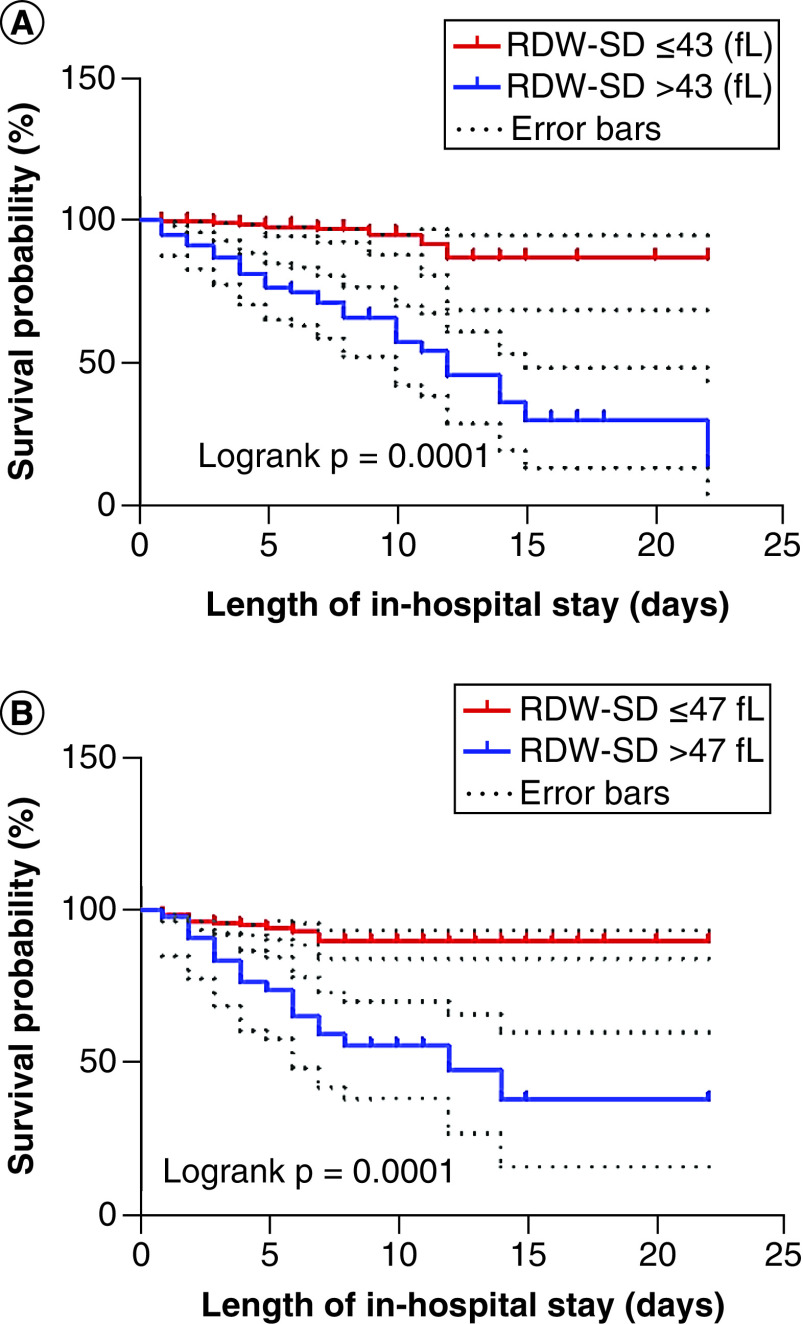
Kaplan-Meier survival curves according to the RDW-SD thresholds of 43 fl (A) and 47 fl (B). The survival probability (%) was significantly lower in the patients with RDW-SD >43 fl and/or >47 fl than in those with RDW-SD ≤43 fl and/or ≤47 fl, respectively. RDW-SD: Standard deviation of red blood cell distribution width.

After 2-months follow-up of 331 COVID-19 patients with various length of in-hospital stay (6.40 ± 4.048 days), 40 patients (10.1%) had died in a mean duration of 4.37 (2.94) days. The survival probability % was significantly lower in the patients with RDW-SD >43 fl (62.79%) and/or >47 fl (56.82%) than in those with RDW-SD ≤43 fl (96.73%) and/or ≤47 fl (92.68%), respectively (log-rank: p = 0.0001; Figure 3). The hazard ratio for the mentioned RDW-SD thresholds were respectively 2.736 (95% CI 1.698–4.409; p = 0.0001) and 2.563 (95% CI 1.472–4.465; p= 0.001).

The univariate Cox model was performed to describe the severity and mortality associated biomarkers in COVID-19 patients, which may be confounders for the predictive value of RDW-SD. These significant markers include age, neutrophils, lymphocyte, RDW-CV, direct bilirubin, BUN, serum creatinine and LDH. Gender and some other biomarkers did not have a significant hazard ratio, and so were deleted from the multivariate model. Also, the multicollinearity was analyzed by considering a cut-off value of 10 for variance inflation factor (VIF), in other words, the variables with VIF >10 meet a high multicollinearity, which have been deleted from the multivariate model to correct the multicollinearity in Cox regression (Tables 3 & 4).

**Table 3.** Univariate and multivariable Cox model with all significant COVID-19 biomarkers as well as age for prediction of disease severity (severe: 1; nonsevere: 0) based on red cell distribution width-standard deviation (fl) thresholds.

Variables	Univariate			Multivariate analysis with all significant variables	
	HR (95% CI)	p-value	VIF	Adjusted HR (95% CI)	p-value
Gender	1.316 (0.877–1.974)	0.18	1.182	–	–
Age	1.025 (1.012–1.038)	^†^	1.310	1.010 (0.997–1.024)	0.13
Neutrophils (%)	1.050 (1.030–1.070)	^†^	8.963	0.996 (0.956–1.038)	0.84
Lymphocyte (%)	0.939 (0.919–0.960)	^†^	9.446	0.953 (0.909–0.998)	0.04
Hb (g/dl)	0.918 (0.827–1.018)	0.10	17.871	–	–
Hct (%)	0.978 (0.943–1.014)	0.23	15.941	–	–
MCV (Fl)	1.016 (0.988–1.045)	0.26	1.792	–	–
RDW-CV (%)	1.090 (1.017–1.168)	0.01	1.732	0.807 (0.675–0.965)	0.02
ESR (mm/h)	1.003 (0.996–1.010)	0.36	1.199	–	–
Direct bilirubin	1.462 (1.213–1.762)	^†^	1.177	1.230 (0.977–1.548)	0.08
BUN, serum (mg/dl)	1.021 (1.014–1.027)	^†^	2.257	1.017 (1.007–1.028)	^‡^
Creatinine (mg/dl)	1.168 (1.060–1.287)	^‡^	1.872	0.909 (0.755–1.094)	0.31
LDH (units/l)	1.001 (1.001–1.002)	^‡^	1.113	1 (1–1.001)	0.20
Continuous RDW-SD	1.058 (1.041–1.076)	^†^	2.701	1.080 (1.047–1.115)	^†^
				**Multivariate analysis with age**	
Age Continuous RDW-SD	1.058 (1.041–1.076)	^†^		1.017 (1.004–1.031) 1.052 (1.033–1.071)	^‡^ ^†^
Threshold ≤43 vs >43	1.913 (1.579–2.317)	^†^		1.796 (1.382–2.333)	^†^
Threshold ≤47 vs >47	2.006 (1.608–2.501)	^†^		1.721 (1.192–2.485)	^†^

† p-value < 0.001.

‡ p-value < 0.01.

BUN: Blood urea nitrogen; ESR: Erythrocyte sedimentation rate; Hb: Hemoglobin; Hct: Hematocrit; HR: Hazard ratio; LDH: Lactate dehydrogenase; MCV: Mean corpuscular volume; RDW-CV: Coefficient of variation of red blood cell distribution width; RDW-SD: Standard deviation of red blood cell distribution width; VIF: Variance inflation factor.

**Table 4.** Univariate and multivariable Cox model with all significant COVID-19 biomarkers as well as age for prediction of survival in COVID-19 patients based on red cell distribution width-standard deviation (fl) thresholds.

Variables	Univariate			Multivariate analysis with all significant variables	
	HR (95% CI)	p-value	VIF	HR (95% CI)	p-value
Gender	1.445 (0.761–2.741)	0.26	1.182	–	–
Age	1.044 (1.022–1.065)	^†^	1.310	1.030 (1.005–1.056)	0.02
Neutrophils (%)	1.056 (1.024–1.088)	^†^	8.963	0.987 (0.936–1.040)	0.61
Lymphocyte (%)	0.920 (0.887–0.954)	^†^	9.446	0.947 (0.890–1.009)	0.09
Hb (g/dl)	0.852 (0.727–0.999)	0.05	17.871	–	–
Hct (%)	0.960 (0.908–1.015)	0.15	15.941	–	–
MCV (Fl)	1.045 (1.001–1.090)	0.04	1.792	–	–
RDW-CV (%)	1.132 (1.048–1.223)	^‡^	1.732	0.807 (0.675–0.965)	0.02
ESR (mm/h)	1.001 (0.989–1.013)	0.85	1.199	–	–
Direct bilirubin	1.781 (1.434–2.212)	^†^	1.177	1.614 (1.184–2.202)	^‡^
BUN, serum (mg/dl)	1.027 (1.019–1.035)	^†^	2.257	1.016 (1.002–1.031)	0.02
Creatinine (mg/dl)	1.218 (1.082–1.370)	^‡^	1.872	0.938 (0.731–1.203)	0.61
LDH (units/l)	1.002 (1.001–1.003)	^‡^	1.113	1.001 (1.000–1.002)	0.04
Continuous RDW-SD	1.071 (1.049–1.094)	^†^	2.701	1.101 (1.049–1.157)	^†^
				**Multivariate analysis with age**	
Age Continuous RDW-SD	1.071 (1.049–1.094)	^†^		1.023 (1.002–1.044) 1.059 (1.035–1.083)	0.03 ^†^
Threshold ≤43 vs >43	3.084 (2.057–4.623)	^†^		2.736 (1.698–4.409)	^†^
Threshold ≤47 vs >47	3.104 (2.126–4.533)	^†^		2.563 (1.472–4.465)	^†^

† p-value < 0.001.

‡ p-value < 0.01.

BUN: Blood urea nitrogen; ESR: Erythrocyte sedimentation rate; Hb: Hemoglobin; Hct: Hematocrit; HR: Hazard ratio; LDH: Lactate dehydrogenase; MCV: Mean corpuscular volume; RDW-CV: Coefficient of variation of red blood cell distribution width; RDW-SD: Standard deviation of red blood cell distribution width; VIF: Variance inflation factor.

Based on the multivariate Cox analysis results, the estimated hazard ratios for disease severity and mortality associated with RDW-SD levels are not significantly changed by adding the significant COVID-19 markers, indicating these markers are not confounders (Tables 3 & 4). Furthermore, the results of multivariable Cox models showed that the hazard ratios significantly increased with adding categorically coded RDW-SD (43 and 47 thresholds) compared with the only continuously coded RDW-SD (Tables 3 & 4).

## Discussion

This study validates RDW levels as an independent prognostic factor for categorizing COVID-19 patients into severe and nonsevere groups. RDW, a component of complete blood counts reflecting cellular volume variation, has been shown to be associated with elevated risk for mortality in COVID-19 patients [21]. Previously, the strong predictive power of RDW for predicting the risk of mortality and poor outcomes in other infectious and critical illnesses was identified, including hepatitis B virus-related chronic liver diseases [22,23], coronary artery disease [24], acute interstitial pneumonia [25], influenza [19], ARDS [26] and sepsis [27].

Based on an electronic search reporting on three studies on the total number of 11,445 COVID-19 patients and 2654 severe patients, RDW-CV was higher in COVID-19 patients with severe illness than in those with mild disease. They revealed that the absolute RDW-CV value was 0.69% higher in severe patients compared with those with mild disease [28]. A retrospective study has been conducted to investigate the relationship between RDW and COVID-19 mortality risk in 1198 adult patients diagnosed with SARS-CoV-2 at 4 Partners Healthcare Network Hospitals between 4 March 2020, and 28 April 2020. The elevated RDW (>14.5%) was associated with increased mortality in patients of all ages with a risk ratio of 2.5 (95% CI: 2.3–2.8). Stratified by age, the risk ratio was 6.2 (4.4–7.9; n = 312) <50 years, 3.2 (2.5–4.1; n = 230) 50–60, 2.3 (1.6–3.1; n = 236) 60–70, 1.2 (0.7–1.8; n = 203) 70–80 and 1.9 (1.5–2.3; n = 216) >80 years [21].

In another retrospective study, the clinical outcomes of hospitalized COVID-19 patients were evaluated for their RDW values. In-hospital mortality was defined as primary outcome, while septic shock, need for mechanical ventilation and length of in-hospital stay were secondary outcomes. Among a total of 294 COVID-19 patients, prevalence of increased RDW was 49.7% (146/294). RDW was associated with increased risk of in-hospital mortality (OR: 4.5; 95% CI: 1.4–14.3) and septic shock (OR: 4.6; 95% CI: 1.4–15.1) after adjusting for anemia, ferritin and lactate [29].

Generally, our findings are consistent with the previous COVID-19-related reports, but not in detail. Although the RDW-CV (%) levels showed a significant correlation with some biomarkers and may partially reflect the general health status in COVID-19 patients, the AUC for RDW-CV to predict disease severity and death was not acceptable (less than 70%). This may be due to the fact that the high level of MCV affects the RDW-CV and can decrease its levels, whereas RDW-SD (fl) is an independent statistical index and not affected by a high level of MCV. In this regard, RDW-SD could be a screening indicator to identify nonsevere patients and low risk of death and/or vice versa.

To date, several studies on COVID-19 have been performed to identify the disease severity and the corresponding risk of death, yet their results are not comparable to our results. In recent months, Gong *et al.* constructed a risk nomogram model for early identification of patients at high risk of progression to severe COVID-19. After evaluating 189 patients, they showed that age, RDW, BUN, CRP, LDH, ALB and DBIL were predictive factors for severe COVID-19 [3]. They only presented seven easy-access features in their nomogram while our study has presented a broad range of risk factors associated with RDW levels. The level of RDW-SD was significantly higher in patients with base diseases, particularly hypertension and cardiovascular disorders. Moreover, high levels of RDW-SD were notably associated with age and a panel of laboratory risk factors implies that the RDW-SD levels can also reflect the general health status in COVID-19 patients. Although patients with high RDW levels had lower hemoglobin levels, a multivariate analysis showed that only high RDW was associated with mortality. The hazard ratios for disease severity and mortality associated with RDW-SD levels significantly increased with adding categorically coded RDW-SD 43 and/or 47 thresholds compared with the only continuously coded RDW-SD.

In a meta-analysis study, the prevalence of fever was 88.3% in nonsevere subjects and 93.5% in severe subjects [30]. In the present study, the prevalence of fever in nonsevere and severe patients was respectively 75.97 and 93.88%, which was consistent with the previous reports. In our study, dry cough in nonsevere and severe patients were 54.8 and 80.78%, respectively, which were different from the results of a study in China [30]; they reported a prevalence of 66.5 and 71.8% in nonsevere and severe patients, respectively. Also, the prevalence of fatigue in the severe group of this study was more than twice that of the nonsevere group (95.92 vs 46.35%; p = 0.0001) while this difference in Chinese patients was not significant (42.7 vs 52.3%; p = 0.35) [30]. The frequency of severe patients with shortness of breath in this study (58.16 vs 17.6%; p = 0.0001) and meta-analysis study (42.7 vs 16.3%; p < 0.0001) [30] were significantly more than nonsevere patients.

The exact mechanism by which a high RDW level is correlated with poor outcomes in viral infections may be due to deregulation of erythrocytes homeostasis and impaired red blood cell production. Inflammation and oxidative states may cause insufficient erythropoiesis and RBC alteration and deformation. Interactions between several factors, including proinflammatory cytokines (IL-1, TNF-α, IFN-γ, IL-8), erythropoietin and the hematopoietic response to erythropoietin [31–34]. Nutritional deficiency-related anemia arising from iron, folic acid and vitamin B12 deficiency can result in higher RDW levels [35]. The inflammatory interactions, for example, cytokine storm, affect erythropoiesis, the half-life of RBCs, iron metabolism and hemolysis, which lead to impaired hematopoiesis and the heterogeneity of RBCs [26]. In sepsis, the inflammatory cytokines could induce RBC damage, steady-state distribution of iron, downregulation of erythropoietin receptor and bone marrow suppression, which eventually lead to the RDW elevation [36].

A significantly lower level of bicarbonate (HCO_3_-act) in severe patients along with an acceptable pCO_2_ level implies a progression of metabolic acidosis. Such a metabolic acidosis may be due to irregular inflammatory response (cytokine storm), previously described in severe bacterial sepsis [37] and Chhetri *et al.*'s case study [38]. Although an elevation of the WBC count is a nonspecific finding, it contributes to raising lactic acidosis [39].

The higher levels of renal biomarkers (AST, direct bilirubin and BUN) in severe COVID-19 patients are more likely due to other organ involvement and/or more prevalence of kidney disease in severe patients compared with nonsevere patients.

### Study strengths & limitations

The RDW-SD index is an inexpensive, convenient, practical and quantitative screening and predicting tool that can be obtained directly from the routine blood tests. Second, the relatively large sample size of this study provides a powerful statistical confirmation of the clinical efficacy of this standard biomarker. Of course, prospective studies are generally better than retrospective ones as the retrospective design cannot precisely detect whether there is a causal association between RDW and outcomes or not.

However, this study had a few limitations. Medical intervention and/or other stressful conditions during hospitalization may influence the levels of RDW; further research should be done to prove this theory.

## Conclusion

The significant role of erythrocytes in the pathophysiology of COVID-19 was pointed out in a few recent reports. Based on our findings, the RDW index, particularly, RDW-SD coded 43 and 47 thresholds (fl), showed the potential to differentiate non severe from severe patients as well as predict the death of COVID-19 patients. Thus, RDW-SD levels may be an independent predictor of the severity of infection and mortality probability in COVID-19 patients.

## Future perspective

Research on RBC-associated indexes such as RDW has developed our understanding of coronavirus pathophysiology and risk factors of COVID-19. RDW index, particularly RDW-SD, is now identified as a convenient prognostic biomarker for diagnosing the severity of COVID-19 infection as well as predicting the mortality probability. Thus, its clinical usage helps in the timely treatment of patients. Significant relationships have been identified between the RDW index and other coronavirus-related biomarkers implying that it can also reflect the patient's general health status. Thus, a promising idea for the future application of the RDW index is the inexpensive screening of vulnerable populations for emerging viral infections based on their RDW levels, which helps in the timely implementation of preventive management prior to an epidemic. Furthermore, a future need regarding the role of the RDW index in coronavirus pathogenesis is the investigation of the exact mechanism behind the variation of the RBCs volume and size during viral infections, which may lead to the discovery of new targets for treatment.

**Summary points**

- The current study investigated the prognostic potential of red cell distribution width (RDW) in COVID-19 patients.
- The RDW-coefficient of variation (CV) % levels showed a significant correlation with some biomarkers of COVID-19 (p < 0.05), yet the AUC for RDW-CV to predict disease severity and death was not acceptable (less than 70%).
- A multivariate logistic regression analysis of 331 COVID-19 patients for clinical signs and outcomes based on RDW-standard deviation (SD) fl and age showed that RDW-SD fl levels had a strong association with several symptoms (p < 0.05), the disease severity (OR: 1.195 [1.133–1.261]; p = 0.0001), and survival status (OR: 1.124 [1.073–1.178]; p = 0.0001).
- The RDW-CV % does not have any strong association with the disease severity (OR: 1.096 [0.998–1.204]; p = 0.055) as well as the survival status (OR: 1.110 [1.005–1.225], p = 0.04).
- The survival probability % was significantly lower in the patients with RDW-SD >43 fl (62.79%) and/or >47 fl (56.82%) than in those with RDW-SD ≤43 fl (96.73%) and/or ≤47 fl (92.68%), respectively (log-rank p = 0.0001).
- In COVID-19 patients, the RDW-SD ≤43 fl indicates the non severe illness with no risk of death while 43< RDW-SD ≤47 almost indicates severe illness with low mortality risk.
- The RDW-SD ≤47 fl indicates the non severe illness with low mortality risk while RDW-SD >47 indicates severe illness and a high risk of death.
- In conclusion, RDW-SD proved to be independently a potent predictor for the infection severity as well as a prognostic marker for survival in COVID-19 patients.
- A significantly lower level of bicarbonate (HCO_3_-act) in severe patients along with an acceptable pCO_2_ level implies a progression of metabolic acidosis in severe COVID-19 patients.
