# Supplementary material for: Validation of red cell distribution width as a COVID-19 severity screening tool
Source: Future Sci OA. 2021 Apr 20;7(7):FSO712. doi: 10.2144/fsoa-2020-0199 (PMC8056748; doi:10.2144/fsoa-2020-0199)
Supplement: Supplementary file 1 [file fsoa-07-712-s1.docx]

**SUPPLIMENTARY MATERIALS**

| **Table S1**. Multivariate logistic Regression Analysis of 331 COVID-19 patients for clinical signs and outcomes (categorical variables) based on RDW-SD (fL) and Age.   \|  \| **For Fever** \| \| \| \| \| \| \| \| \| --- \| --- \| --- \| --- \| --- \| --- \| --- \| --- \| --- \| \| **Predictor** \| **β** \| **SEβ** \| **Wald’s**  **χ2** \| **df** \| **p** \| **eβ**  **(odds ratio)** \| ***95.0% C.I.for* eβ** \| \| \| Lower \| Upper \| \| Constant \| -1.096 \| 0.933 \| 1.381 \| 1 \| 0.24 \| 0.334 \|  \|  \| \| age \| 0.040 \| 0.010 \| 16.005 \| 1 \| ******* \| 1.041 \| 1.021 \| 1.061 \| \| **RDW-SD (fL)** \| 0.013 \| 0.023 \| 0.285 \| 1 \| 0.59 \| 1.013 \| 0.967 \| 1.060 \| | | | | | | | | |
| --- | --- | --- | --- | --- | --- | --- | --- | --- | --- | --- | --- | --- | --- | --- | --- | --- | --- | --- | --- | --- | --- | --- | --- | --- | --- | --- | --- | --- | --- | --- | --- | --- | --- | --- | --- | --- | --- | --- | --- | --- | --- | --- | --- | --- | --- | --- | --- | --- | --- | --- | --- | --- | --- | --- | --- |
| \|  \| **For Dry cough** \| \| \| \| \| \| \| \| \| --- \| --- \| --- \| --- \| --- \| --- \| --- \| --- \| --- \| \| Constant \| -3.231 \| 0.832 \| 15.062 \| 1 \| ******* \| 0.040 \|  \|  \| \| age \| 0.059 \| 0.009 \| 41.308 \| 1 \| ******* \| 1.061 \| 1.042 \| 1.080 \| \| **RDW-SD (fL)** \| 0.017 \| 0.020 \| 0.720 \| 1 \| 0.39 \| 1.017 \| 0.978 \| 1.058 \| | | | | | | | | |
|  | **For Fatigue*** | | | | | | | |
| Constant | -4.947 | 0.922 | 28.802 | 1 | ******* | 0.007 |  |  |
| age | 0.061 | 0.009 | 42.958 | 1 | ******* | 1.063 | 1.044 | 1.083 |
| **RDW-SD (fL)** | 0.054 | 0.022 | 6.067 | 1 | 0.01* | 1.055 | 1.011 | 1.101 |
| \|  \| **For Sputum production** \| \| \| \| \| \| \| \| \| --- \| --- \| --- \| --- \| --- \| --- \| --- \| --- \| --- \| \| Constant \| -4.025 \| 0.830 \| 23.522 \| 1 \| ******* \| 0.018 \|  \|  \| \| age \| 0.055 \| 0.009 \| 39.579 \| 1 \| ******* \| 1.057 \| 1.039 \| 1.075 \| \| **RDW-SD (fL)** \| 0.033 \| 0.020 \| 2.812 \| 1 \| 0.09 \| 1.034 \| 0.994 \| 1.075 \| | | | | | | | | |
|  | **For Loss of smell*** | | | | | | | |
| Constant | -4.089 | 0.754 | 29.385 | 1 | ******* | 0.017 |  |  |
| age | 0.024 | 0.008 | 8.292 | 1 | ****** | 1.024 | 1.008 | 1.041 |
| **RDW-SD (fL)** | 0.045 | 0.018 | 6.508 | 1 | 0.01* | 1.046 | 1.010 | 1.083 |
| \|  \| **For Shortness of breath** \| \| \| \| \| \| \| \| \| --- \| --- \| --- \| --- \| --- \| --- \| --- \| --- \| --- \| \| Constant \| -2.818 \| 0.701 \| 16.167 \| 1 \| ******* \| 0.060 \|  \|  \| \| age \| 0.014 \| 0.008 \| 3.119 \| 1 \| 0.07 \| 1.014 \| 0.998 \| 1.030 \| \| **RDW-SD (fL)** \| 0.029 \| 0.017 \| 2.932 \| 1 \| 0.08 \| 1.029 \| 0.996 \| 1.064 \| | | | | | | | | |
|  | **For Muscle or joint pain*** | | | | | | | |
| Constant | -4.765 | 0.794 | 36.017 | 1 | ******* | 0.009 |  |  |
| age | 0.030 | 0.008 | 13.979 | 1 | ******* | 1.030 | 1.014 | 1.046 |
| **RDW-SD (fL)** | 0.063 | 0.019 | 11.516 | 1 | ** | 1.066 | 1.027 | 1.105 |
|  | **For Sore throat*** | | | | | | | |
| Constant | -5.516 | 0.892 | 38.223 | 1 | ******* | 0.004 |  |  |
| age | 0.032 | 0.010 | 9.740 | 1 | ****** | 1.032 | 1.012 | 1.053 |
| **RDW-SD (fL)** | 0.049 | 0.019 | 6.364 | 1 | 0.01* | 1.050 | 1.011 | 1.091 |
|  | **For Headache*** | | | | | | | |
| Constant | -5.686 | 0.861 | 43.573 | 1 | ******* | 0.003 |  |  |
| age | 0.031 | 0.009 | 11.430 | 1 | ****** | 1.032 | 1.013 | 1.050 |
| **RDW-SD (fL)** | 0.064 | 0.019 | 11.185 | 1 | ****** | 1.066 | 1.027 | 1.107 |
| \|  \| **For Diarrhoea** \| \| \| \| \| \| \| \| \| --- \| --- \| --- \| --- \| --- \| --- \| --- \| --- \| --- \| \| **Predictor** \| **β** \| **SEβ** \| **Wald’s**  **χ2** \| **df** \| **p** \| **eβ**  **(odds ratio)** \| ***95.0% C.I.for* eβ** \| \| \| Lower \| Upper \| \| Constant \| -1.586 \| 1.375 \| 1.332 \| 1 \| 0.24 \| 0.205 \|  \|  \| \| age \| -0.024 \| 0.015 \| 2.423 \| 1 \| 0.12 \| 0.977 \| 0.948 \| 1.006 \| \| **RDW-SD (fL)** \| 0.002 \| 0.034 \| 0.003 \| 1 \| 0.95 \| 1.002 \| 0.937 \| 1.072 \| | | | | | | | | |
|  | **For Survival status***** | | | | | | | |
| **Predictor** | **β** | **SEβ** | **Wald’s**  **χ2** | **df** | **p** | **eβ**  **(odds ratio)** | ***95.0% C.I.for* eβ** | |
|  |  |  |  |  |  |  | Lower | Upper |
| Constant | -9.234 | 1.231 | 56.299 | 1 | ******* | 0.0001 |  |  |
| age | 0.037 | 0.012 | 8.937 | 1 | ****** | 1.037 | 1.013 | 1.063 |
| **RDW-SD (fL)** | 0.117 | 0.024 | 24.203 | 1 | ******* | 1.124 | 1.073 | 1.178 |
|  | **For Severity of Disease (severe: 1; non-severe: 0)** *** | | | | | | | |
| Constant | -9.753 | 1.177 | 68.665 | 1 | ******* | 0.0001 |  |  |
| age | 0.027 | 0.009 | 8.497 | 1 | ****** | 1.028 | 1.009 | 1.046 |
| **RDW-SD (fL)** | 0.179 | 0.027 | 42.964 | 1 | ******* | 1.195 | 1.133 | 1.261 |
|  | **For Basic disease*** | | | | | | | |
| Constant | -0.883 | 0.708 | 1.559 | 1 | 0.21 | 0.413 |  |  |
| age | -0.003 | 0.007 | 0.185 | 1 | 0.66 | 0.997 | 0.983 | 1.011 |
| **RDW-SD (fL)** | 0.038 | 0.018 | 4.294 | 1 | 0.03* | 1.038 | 1.002 | 1.076 |
| *** P-Value < 0.001  ** P-Value < 0.01  **Table S2**. Multivariate logistic Regression Analysis of 331 COVID-19 patients for outcomes (categorical variables) based on **RDW-CV (%)** and Age. | | | | | | | | |
|  | **For Survival status** | | | | | | | |
| **Predictor** | **β** | **SEβ** | **Wald’s**  **χ2** | **df** | **p** | **eβ**  **(odds ratio)** | ***95.0% C.I.for* eβ** | |
|  |  |  |  |  |  |  | Lower | Upper |
| Constant | -6.619 | 1.059 | 39.095 | 1 | ******* | 0.001 |  |  |
| age | 0.052 | 0.012 | 19.151 | 1 | ******* | 1.053 | 1.029 | 1.078 |
| **RDW-CV (%)** | 0.104 | 0.051 | 4.230 | 1 | 0.04 | 1.110 | 1.005 | 1.225 |
|  | **For Severity of Disease (severe: 1; non-severe: 0)** | | | | | | | |
| Constant | -4.605 | 0.805 | 32.690 | 1 | ******* | 0.010 |  |  |
| age | 0.042 | 0.008 | 25.549 | 1 | ******* | 1.043 | 1.026 | 1.061 |
| **RDW-CV (%)** | 0.092 | 0.048 | 3.695 | 1 | 0.05 | 1.096 | 0.998 | 1.204 |

*** P-Value < 0.001

** P-Value < 0.01
